# Utility of Contact Impedance Mapping in Differentiating the Mechanism of Focal Atrial Tachycardia

**DOI:** 10.19102/icrm.2019.100501

**Published:** 2019-05-15

**Authors:** Sumeet K. Mainigi, Allan M. Greenspan

**Affiliations:** ^1^Section of Electrophysiology, Division of Cardiovascular Disease, The Institute for Heart and Vascular Health, Einstein Medical Center, Philadelphia, PA, USA

**Keywords:** Contiguous low-impedance area, electrogram characteristics, impedance map, source-sink shunting, triggered activity

## Abstract

Contact impedance mapping can differentiate focal atrial tachyarrhythmias from macroreentry (atrial flutter) and localized reentry (atrioventricular nodal reentry tachycardia) by detecting different patterns of regional unipolar tissue impedance distribution. Specifically, focal atrial tachycardia (AT) is characterized by the finding of a contiguous low-impedance area (CLIA) adjacent to the site of origin, surrounded by normal tissue impedance levels. However, it remains unclear whether or not this finding could distinguish different mechanisms of focal AT. In the present study, we sought to determine whether impedance and voltage maps in patients with microreentrant AT differ from those created due to triggered activity. Consecutive patients undergoing electrophysiologic study and the ablation of AT were included. All patients underwent mapping and ablation procedures in a standard manner. Contact impedance and voltage maps were collected in the background and analyzed offline for comparison. A total of 50 patients with 75 focal ATs were studied and ablated, and the mechanism of AT (ie, triggered activity versus microreentry) was determined. The 41 ATs attributed to triggered activity in 30 patients all demonstrated a CLIA containing or adjacent to the successful ablation site, while the 34 ATs in the 20 patients attributed to microreentry demonstrated uniform impedance. In contrast, microreentrant AT patients were more likely to have scar located adjacent to the site of origin (88.9% versus 18.2%). Three-dimensional mapping employing both contact impedance mapping and voltage mapping can reliably identify the mechanism of focal AT.

## Introduction

Contact impedance mapping has been used to differentiate focal atrial tachyarrhythmia from macroreentry (atrial flutter)^1^ and localized reentry (atrioventricular nodal reentrant tachycardia)^2^ by detecting different patterns of impedance, presumably reflecting the differences in the regional patterns of perimembrane and transmembrane current flow seen with each of these arrhythmia mechanisms. Specifically, the finding of a contiguous low-impedance area (CLIA) surrounded by normal impedance, of a consistent surface area of 3.0 cm^2^ ± 2.0 cm^2^, occurs only in focal atrial tachycardia (AT) and not in the other mechanisms.^1^ We hypothesized that the existence of this CLIA may be due to a higher density of current flow in a localized region due to the afterdepolarizations that cause triggered activity and which drive the membrane to threshold in addition to the current resulting from the regenerative action potential. Importantly, since the majority of focal ATs are caused by triggered activity,^3^ this would explain the ubiquity of this finding in association with focal AT.

Theoretically, a microreentrant circuit activated at a high enough frequency could also produce a low-impedance area, but such might require a rotor being activated at greater than 10 Hz and would likely result in atrial fibrillation rather than a focal AT. There is, however, a class of focal ATs that are adenosine-insensitive and which have electrogram characteristics at the site of origin that are suggestive of microreentry.^3^ If the CLIA seen in focal AT results only from triggered automaticity, it should be absent in such microreentrant ATs.

We therefore studied 27 consecutive patients with microreentrant ATs with local activation time (LAT), voltage, and impedance maps and compared them to a cohort of 33 consecutive patients with focal ATs caused by triggered activity (five patients had both mechanisms of focal AT) in the present study. Our aim was to define the impedance map characteristics of the microreentrant ATs and determine whether or not there exist differences in comparison with the impedance maps of those with triggered ATs.

## Methods

### Patient selection

Fifty-five consecutive patients undergoing electrophysiologic study and ablation for suspected focal AT at the Einstein Medical Center in Philadelphia, PA, between March 2008 and June 2015 were included in this study.

### Electrophysiologic study and the acquisition of local activation time, voltage, and impedance data

After obtaining written informed consent approval from the Albert Einstein Medical Center Institutional Review Board, all patients underwent electrophysiologic study using a 4-mm-tip EZ Steer Thermocool or SmartTouch mapping and ablation catheter (Biosense Webster, Diamond Bar, CA, USA) and the Carto^®^ XP or Carto^®^ 3 electroanatomic mapping system (Biosense Webster, Diamond Bar, CA, USA). Three-dimensional electroanatomic maps were generated by translocating the mapping catheter to between 87 points and 363 points (mean: 176.3 ± 63 points) throughout the chamber of interest. In addition to LAT and voltage maps, tissue impedance data were obtained at each site by passing a 50-Hz, 10-μA current through the ablation catheter into the tissue. The voltage drop was measured, and the tissue impedance was calculated and annotated at each point on the three-dimensional electroanatomic shell. The calculated tissue impedance was based on unipolar voltage measurements between the catheter tip and a ground patch on the patient’s posterior thoracic wall. Mapping point densities were calculated and compared among patients to ensure consistency between maps.

As previously reported in our laboratory protocol,^1^ we measured the minimum and maximum tissue impedances for the chamber of interest and operationally defined low impedance as less than or equal to the minimum impedance plus 10% of the impedance range [ie, Z_low_ ≤ Z_min_ + 0.1(Z_max_ − Z_min_)] and normal impedance as greater than or equal to the minimum impedance plus 20% of the impedance range [ie, Z_norm_ ≥ Z_min_ + 0.2(Z_max_ − Z_min_)]. We dichotomized the impedance map color scale such that low impedance was denoted by red and normal impedance by purple, respectively. Impedance measurements that were markedly elevated because they were measured in tissue with associated low-volume blood pools (eg, the coronary sinus, vena cavae, pulmonary veins) were excluded. The mean tissue impedance for each impedance map in 12 patients with triggered-firing AT and six patients with microreentrant AT were analyzed, as shown in **Table 1**.

Standard electrophysiologic pacing maneuvers were used to identify the mechanism of the supraventricular tachycardia (SVT). In all 55 patients, the mechanism of the 75 SVTs (several patients had more than one SVT) was confirmed to be focal AT by the three-dimensional LAT map displaying a centrifugal activation pattern and by successful focal ablation at the site of earliest local activation demonstrating sharp, completely negative unipolar electrograms, respectively.

Further analysis of the arrhythmia mechanism was performed by studying the bipolar electrogram characteristics at the site of origin, regarding electrogram voltage amplitude, duration, and fractionation. Sharp, normal-amplitude (> 1.5 mV), and normal-duration (< 70 ms) electrograms were considered suggestive of triggered activity, while low-amplitude (< 0.5 mV) and long-duration (> 100 ms) electrograms with fractionation, split potentials, or regions showing continuous electrical activity, near the effective ablation site, were considered to be suggestive of microreentry.

Impedance maps were analyzed retrospectively for the presence or absence of CLIAs and their anatomic localization in relation to the successful ablation site. Additionally, bipolar voltage maps were also analyzed for the presence, location, and extensiveness of scarring.

### Statistical analysis

Descriptive statistics were reported as means ± standard deviations for continuous variables that were normally distributed. The comparison of continuous variables (eg, impedance, voltage amplitude, point densities, cycle lengths) between distinct patients was evaluated using two-tailed, unpaired Student’s t-tests. To account for the disproportionate contribution of impedance measurements from the five patients with both AT mechanisms, weighted analyses were performed. To further confirm the findings, sensitivity analyses were completed that included only the remaining 55 patients who contributed single impedance datasets. Group comparisons of categorical variables were evaluated using the chi-squared test. A two-tailed p value of less than 0.05 was considered to be statistically significant.

## Results

### Patient demographics

There were 23 males (42%) and 32 females (58%) in the study cohort, with a total of 75 focal ATs studied and ablated. The mean age of the cohort was 64.7 years ± 14.3 years. Forty-eight patients (96%) had hypertension, 26 patients (52%) had diabetes mellitus, 15 patients (30%) had coronary artery disease, and 12 (24%) had previous CVAs. Sixteen patients (32%) were smokers. The mean left ventricular ejection fraction was 44.9% ± 13.2%.

### Diagnostic criteria for focal atrial tachycardia

All 75 SVTs studied in the 55 patients showed three-dimensional LAT maps with a centrifugal pattern indicative of focal AT. Termination of right ventricular entrainment during SVT demonstrated the classic VAAV response seen in focal AT in all 75 SVTs. The LAT maps showed that the total activation time of the SVT was less than 70% of the atrial cycle length, arguing against macroreentry as the SVT mechanism. Forty-nine of the focal ATs originated from the right atrium and 26 originated from the left atrium. All 75 SVTs required either programmed atrial stimulation or burst right atrial pacing at cycle lengths of 300 ms to 200 ms with or without isoproterenol for induction.

### Evidence for the mechanism of the focal atrial tachycardia

#### Triggered activity

**Figure 1:**
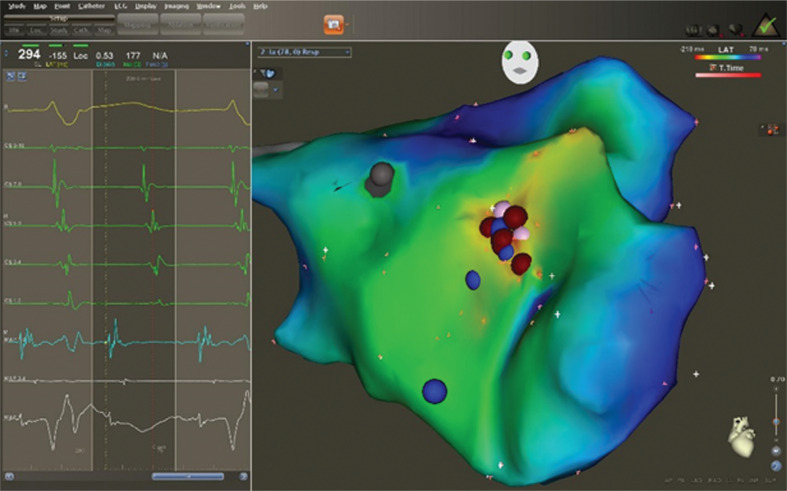
Intracardiac electrograms, including that from the site of origin (maps 1 and 2) of a focal left AT emanating from the superolateral mitral annular region, along with the corresponding three-dimensional LAT electroanatomic map. Note the sharp, normal-amplitude (> 1.0 mV), short-duration (< 60 ms) electrogram (maps 1 and 2) from the successful ablation site (red dots) and the centrifugal activation pattern of the LAT map, consistent with a triggered-firing focal AT.

Forty-one (55%) of the 75 focal ATs in 33 patients had sharp, short-duration (< 75 ms), normal-amplitude (> 1.0 mV) bipolar electrograms recorded at or near the site of origin, defined by the earliest LATs. They also demonstrated sharp, completely negative unipolar electrograms that were within 5 mm of the successful ablation site. Eight patients were tested with entrainment, and all showed long postpacing intervals (PPIs; > atrial cycle length + 30 ms). Three additional patients were tested for adenosine responsiveness and all three ATs terminated with 12 mg of intravenous adenosine. The electrogram duration measured at the site of origin was an average of three measurements. The mean electrogram duration for these 41 focal ATs was 58.8 ms ± 13.4 ms. This electrogram duration comprised 15.5% ± 6.8% of the atrial cycle length. **Figure 1** shows the electrogram recordings and an LAT map from a right atrial focal AT due to triggered activity.

#### Microreentry

The other 34 focal ATs in the remaining 27 patients showed highly fractionated, low-amplitude (< 0.5 mV), long-duration (; 100-ms) electrograms at the site of origin. Entrainment in six patients demonstrated short PPIs (ie, atrial cycle length + 30 ms). An additional patient received 12 mg of intravenous adenosine with no effect on the focal AT. In nine other patients, electrogram recordings from the site of origin showed continuous electrical activity indicative of localized reentry.

**Figure 2:**
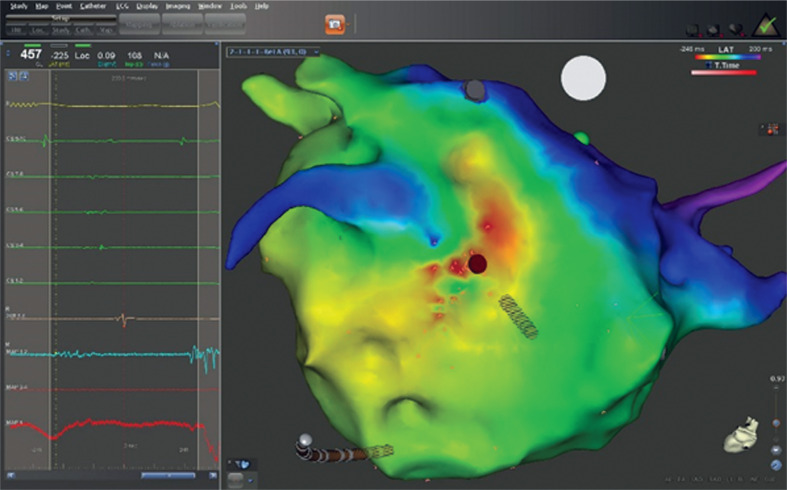
Intracardiac electrograms, including that from the site of origin (maps 1 and 2) of a microreentrant left AT emanating from the midposterior left atrial wall (red dot), along with the corresponding three-dimensional LAT electroanatomic map. Note the highly fractionated, low-amplitude (< 0.5 mV), very-long-duration (> 95 ms) electrogram from the successful ablation site (red dot) on the LAT map.

The mean electrogram duration in these 34 focal ATs was 162.5 ms ± 13.4 ms and composed 47% ± 12% of the atrial cycle length. **Figure 2** shows the highly fractionated electrograms and an LAT map from a left atrial microreentrant focal AT.

Based on the electrogram characteristics and patient responses to entrainment and adenosine, we classified these two distinct groups of focal ATs as (1) ATs resulting from triggered activity (ie, ATs with short-duration, normal-amplitude electrograms with negative entrainment and adenosine sensitivity) or (2) microreentrant ATs (ie, ATs with highly fractionated, long-duration, low-amplitude electrograms with positive entrainment and adenosine unresponsiveness).

### Impedance map findings in focal atrial tachycardias

The contact impedance map in all 41 focal ATs with short-duration, normal-amplitude, sharp electrograms revealed a CLIA either containing the successful ablation site or an area within 1.5 cm of it. The mean surface area of the CLIA was 3.23 cm^2^ ± 1.6 cm^2^.

**Figure 3:**
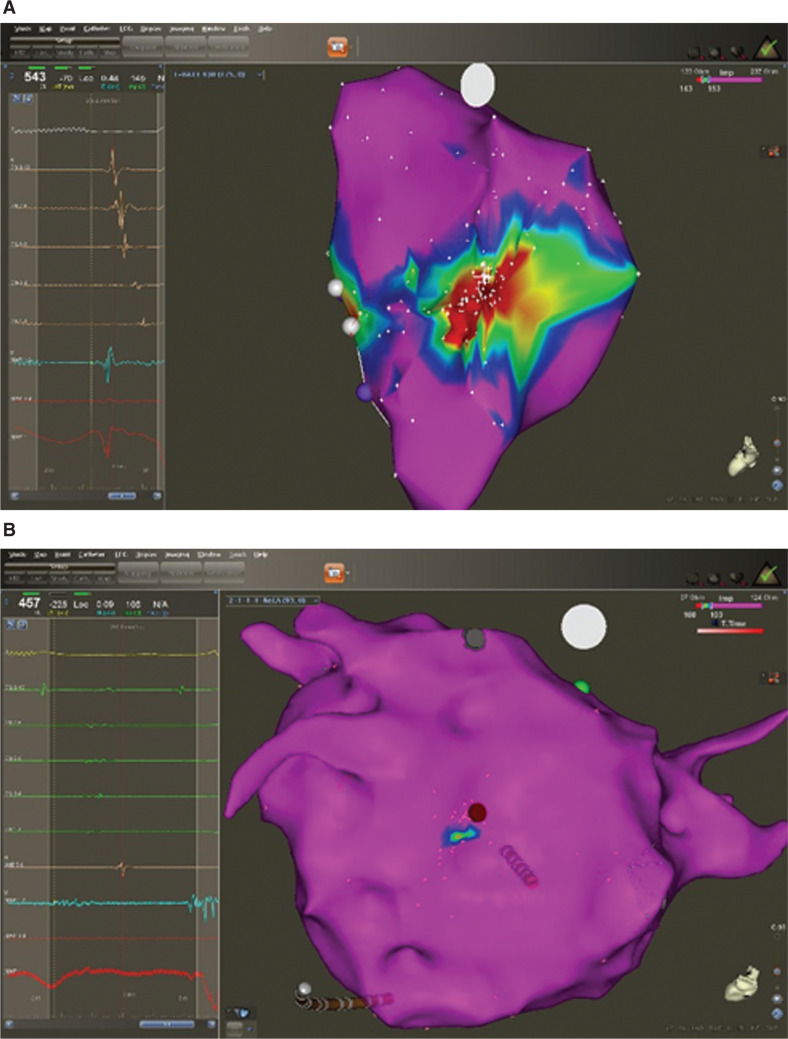
**A:** Intracardiac electrograms, including that from the site of origin (maps 1 and 2) of a triggered-firing focal left AT emanating from the midposterior left atrial wall (red dot) as well as the corresponding three-dimensional electroanatomic impedance map. Note the sharp, normal-amplitude, short-duration electrograms from the site of origin (maps 1 and 2) and the finding of a CLIA indicated by the red area surrounding the successful ablation site (red dot) in the impedance map. **B:** Intracardiac electrograms, including that from the site of origin (maps 1 and 2) of a microreentrant focal left AT emanating from the midposterior left atrial wall (red dot) along with the three-dimensional electroanatomic impedance map. Note the highly fractionated, low-amplitude, long-duration intracardiac electrogram (maps 1 and 2) from the successful ablation site (red dot). Note also, in contrast with the triggered-firing AT impedance map, there is no CLIA containing or adjacent to the successful ablation site seen in the microreentrant impedance map.

In the 34 focal ATs with long-duration, low-amplitude fractionated electrograms at the site of origin, the impedance maps showed a uniform pattern of normal impedance with no CLIAs. These impedance map findings in the two different focal ATs are presented in **Figure 3**.

Of note is the fact that no CLIA was observed in the impedance maps generated during sinus rhythm in 25 patients undergoing electrophysiologic study and mapping/ablation for AT and atrial flutter. Nor was a CLIA observed in five patients who also had impedance maps generated during rapid, high-right-atrium pacing at the cycle length of the AT or atrial flutter.

### Voltage map findings in focal atrial tachycardias

Bipolar voltage maps were obtained in all 55 patients during the 75 focal ATs. Scar was defined as a voltage of less than 0.3 mV.

Scar adjacent to the site of origin, defined as that within 20 mm, was found in six (18.2%) of the 33 patients with short-duration, normal-amplitude, sharp electrograms. In these patients, the mean scar surface area was 3.9 cm^2^ ± 3.3 cm^2^.

**Figure 4:**
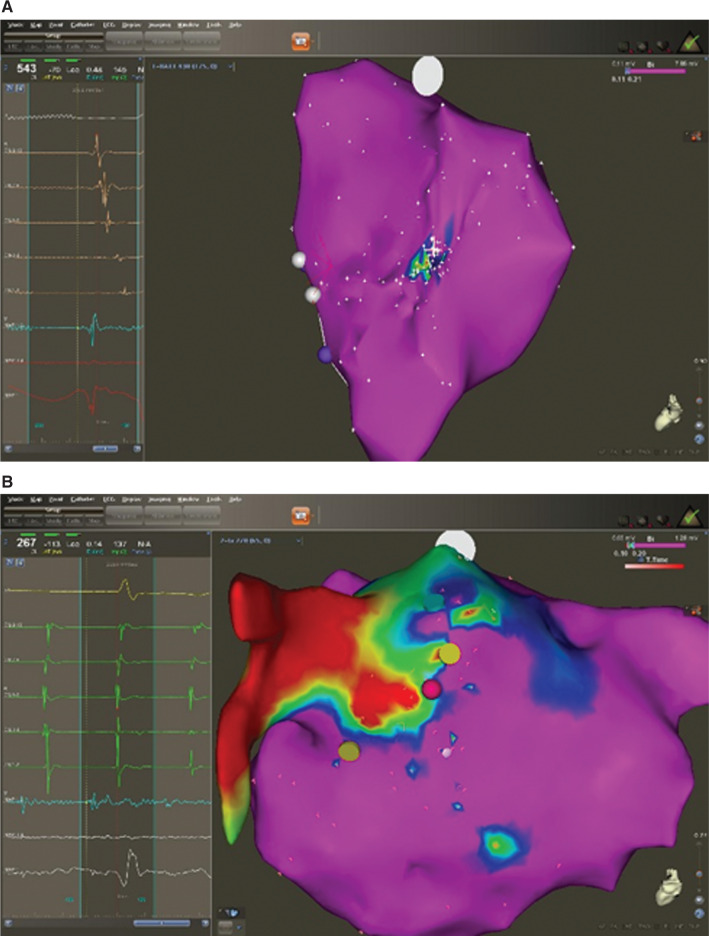
**A:** Intracardiac electrograms, including that from the site of origin (maps 1 and 2) of a triggered-firing focal left AT, emanating from the midposterior left atrial wall and its corresponding bipolar voltage map. Note the sharp, normal-amplitude, short-duration electrogram (maps 1 and 2) from the site of origin (white cross) and the absence of any low-voltage (< 0.11-mV) regions adjacent to the successful ablation site compatible with scar tissue. **B:** Intracardiac electrograms, including that from the site of origin (maps 1 and 2) of a microreentrant focal left AT emanating from the superior posterior left atrial wall close to the origin of the left common pulmonary veins, along with the corresponding three-dimensional electroanatomic voltage map. Note the highly fractionated, low-amplitude, long-duration intracardiac electrogram (maps 1 and 2) from the successful ablation site (red dot) and the presence of an extensive low-voltage (< 0.1 mV) area adjacent to the site of origin, compatible with scar tissue.

In contrast, of the 27 patients with fractionated, long-duration, low-amplitude electrograms at the site of origin, 24 (88.9%) had scar located adjacent to the site of origin, with a mean scar surface area of 6.2 cm^2^ ± 4.9 cm^2^. The difference in scar surface area between triggered-firing ATs and microreentrant ATs was not significant (p = 0.5). Voltage map findings for the two different focal ATs are shown in **Figure 4**.

Five patients had both types of focal AT. When these patients were in the triggered-activity AT state, only two (40%) had scarring adjacent to the site of origin of the AT. However, when the five patients were in the microreentrant AT state, four of the five (80%) had adjacent scarring. This echoed the pattern of the prevalence of scarring adjacent to the site of origin in the two types of focal AT in the entire study cohort.

## Discussion

Impedance mapping has been shown to differentiate focal ATs from macroreentrant^1^ and localized reentrant arrhythmias^2^ via the identification of a CLIA in the impedance map recorded only in the context of focal ATs. There is suggestive evidence that the CLIA is associated with triggered activity. First, there is the occurrence of CLIAs in the impedance maps of outflow tract premature ventricular complexes, which are almost uniformly due to triggered firing. Second, there is the consistent surface area of the CLIA in focal ATs (3.23 ± 1.6 cm^2^), which closely correlates with that of the critical mass of atrial cells predicted by mathematical modeling (n = 300,000) to be necessary to overcome the “source-sink” shunting effect of the atrial syncytium and thereby drive the atrial rhythm.^5^ Third, there is the finding that the surface area of the CLIA in outflow tract premature ventricular complex impedance maps (5.9 ± 3.8 cm^2^) is larger than that in focal ATs, reflecting both the larger surface area of ventricular myocytes and the larger number of myocytes necessary to overcome the “source-sink” shunting of the ventricular syncytium.

Furthermore, the CLIAs associated with outflow tract premature ventricular complex impedance maps have a consistent surface area (5.9 ± 3.8 cm²),^4^ which correlates with the larger surface area of ventricular myocytes (on average, four times that of atrial myocytes) and the larger number of ventricular myocytes necessary (as a result of the greater wall thickness of the ventricle) to form the critical mass (n = 700,000 cells) needed to overcome the syncytial shunting.

We therefore sought to test this hypothesis by determining whether the impedance maps of patients with microreentrant ATs were significantly different from those of triggered AT and thus could be used to differentiate these two arrhythmia mechanisms from one another. We also sought to determine the role of scarring in these two arrhythmia mechanisms and to see if the differences identified in the voltage maps could also be useful in this differentiation.

### Relation of the CLIA to the focal atrial tachycardia mechanism

We used standard techniques to determine the mechanism of focal AT including the response to entrainment, adenosine, and electrogram characteristics at the site of origin. As per Markowitz et al.,^3^ fractionated, low-amplitude (< 0.39 mV), long-duration (> 86 ms) electrograms at the site of origin that make up 44% ± 20% of the AT cycle length correlated with adenosine insensitivity and positive entrainment, thereby establishing the mechanism as microreentry within the limitations of in vivo measurements. Conversely, normal-amplitude (> 0.9 mV), normal-duration (< 70 ms) electrograms at the site of origin, which compose 12% ± 4% of the AT atrial cycle length, correlated with negative entrainment and adenosine responsiveness, consistent with triggered activity.

We found that CLIAs were observed only in the impedance maps of those ATs with normal-amplitude (> 0.9 mV), short-duration (< 70 ms) electrograms at the site of origin as well as those with negative entrainment and positive adenosine sensitivity, which were all compatible with triggered activity. Conversely, no CLIAs were observed in the impedance maps of those focal ATs that had highly fractionated, low-amplitude (< 0.4 mV), long-duration (> 90 ms) electrograms and/or with a positive entrainment response and adenosine insensitivity, which was compatible with the finding of a microreentrant mechanism.

### Relation of scarring to the focal atrial tachycardia mechanism

Although scarring can facilitate triggered activity by reducing intercellular connectivity,^5,6^ decreasing the critical mass of cells in a triggered focus necessary to overcome the “source-sink” shunting effect of the atrial syncytium,^5^ the occurrence of scarring adjacent to the site of origin of a triggered AT is rare. In our patient cohort, such was found in only 18% of cases with triggered activity. Conversely, the prevalence of scarring adjacent to the site of origin of microreentrant AT was much greater than that in triggered-firing AT, being observed in 88.9% of patients. This observation is not that surprising, given the important role that scarring can play in the generation of reentry by providing regions of slow conduction^6–8^ and unidirectional block.

### Possible mechanism of the contiguous low-impedance area

Our current observations demonstrate that the CLIA is found only in the impedance map of focal ATs caused by triggered activity, which are due to afterdepolarizations, and not in those caused by microreeentry wherein activation is generated by the longitudinal circuit current.

There are many factors, theoretically, that could contribute to the formation of the CLIA including rapid activation rates, anatomic factors such as dense scarring or crisscrossing myofibrillar bundles, localized increases in transmembrane current flow, or expansions in intercellular connectivity localized to the region of triggered firing. It is also possible that the CLIA is unrelated to the arrhythmia itself but is a characteristic of patients with focal AT and would be found also during sinus rhythm.

Importantly, we do have some evidence for which factors do not contribute to CLIA formation. First, higher activation rates do not contribute to CLIA formation as indicated by our observation that macroreentrant atrial flutters on average have much shorter atrial cycle lengths than focal ATs, yet do not demonstrate CLIAs.^1^ Also, the observation that rapid atrial pacing at cycle lengths identical to those of triggered-firing focal ATs do not generate CLIAs^1^ suggests that other factors besides activation rate that are specific to triggered activity generate the CLIA. Second, scar tissue, which is known to have significantly lower tissue impedance values than normal myocardium,^9^ does not contribute to formation of the CLIA. This statement is based on our observation of a very low prevalence (18%) of scar tissue adjacent to the site of origin of the focal AT due to triggered activity. Furthermore, this is reinforced by the observation that, in patients with both triggered-firing AT and macroreentrant atrial flutter, CLIAs are observed only in the impedance maps during an AT.^1^ This also indicates that the presence of the CLIA is not structural in origin but rather is related to the physiology of the arrhythmia.

Finally, the occurrence of the CLIA in patients with focal AT is clearly related to the arrhythmia, as it is not found in sinus rhythm maps but rather only in the maps created during the arrhythmia.^1^

Beyond these conclusions, we can only speculate that the generation of the CLIA in triggered firing might be due to factors that increase localized transmembrane current flow in the cells in the region of the CLIA, thereby lowering the resistive component of the local tissue impedance, and/or due to other factors that increase local gap junction connectivity^10–12^ in the cells undergoing triggered firing, thereby lowering local capacitive impedance. A better understanding of which factors related to triggered activity cause the observed CLIA will require data gleaned from in vitro and in vivo animal experiments.

## Limitations

A significant limitation of the study involves the use of a large-surface-area electrode-mapping catheter (3.5 mm), part of whose electrode tip is suspended in the blood pool, which introduces a shunt conductance that reduces the accuracy of the tissue impedance measurement by 67%. However, the shunt conductance throughout the atrial chamber is fairly uniform and should not obviate the ability to detect the relative differences in tissue impedance at the site of triggered activity versus the rest of the chamber not activated by the triggered firing.

A second limitation is the artifact in tissue impedance measurement introduced by poor tissue contact, which gives a falsely low value of tissue impedance. This was more of a problem in conjunction with the early tissue impedance maps made before the advent of force-sensing ablation catheters. In those cases, we deleted mapping points with voltage amplitudes of less than 0.05 mV to avoid this artifact. However, most of the impedance maps were made with the newer force-sensing catheters, thus overcoming this limitation.

A third limitation is the fact that adenosine sensitivity is not a foolproof means of differentiating triggered activity as the mechanism of a focal AT from microreentry. There are some adenosine-sensitive microreentrant focal ATs, but they constitute a very small percentage of these ATs and emanate from specific locations in the atria that contain perinodal tissue whose action potentials are Ca^+2^ channel–dependent and which are usually found in the per-cristae and septal tricuspid annular regions. Since many of our focal ATs did not arise from these locations, false positives for adenosine sensitivity were not a real issue.

A fourth limitation is that we could not directly rule out a role for specific anatomic characteristics in determining the generation of the CLIA, rather than the physiologic changes due to the triggered activity. Although the tissue thickness, the presence of scarring or extensive fibrosis, and/or the amount of local arteriolar blood flow around the CLIA can contribute to localized lower tissue impedance, we can deduce from a number of observations that they are generally not critical factors in the formation of the CLIA as noted.

## Conclusion

In summary, with this study, we have extended the use of contact impedance mapping to analyzing the mechanism of focal ATs. We have shown that the two mechanisms of focal AT, triggered activity and microreentry, are associated with distinct patterns of tissue impedance: specifically, triggered-activity AT shows a CLIA with a consistent surface area of 3.0 cm^2^ ± 2.0 cm^2^, while microreentrant AT shows a uniform impedance pattern without a CLIA.

Furthermore, these two different AT mechanisms have a markedly different prevalence of associated scarring as indicated by the bipolar voltage maps, with the prevalence of adjacent scarring being significantly greater in the microreentrant ATs than in the triggered-activity ATs.

## Figures and Tables

**Table 1:** Comparison of Mean Chamber Tissue Impedance Values Measured During Focal AT in 18 Patients

Triggered AT (n = 12) (Ω)	Microreentrant AT (n = 6) (Ω)
122.2 ± 12.16	119.44 ± 7.7
181.96 ± 9.54	116.18 ± 12.48
177.55 ± 10.04	141.06 ± 8.81
117.96 ± 9.13	152.90 ± 23.67
135.64 ± 6.86	140.05 ± 7.98
160.12 ± 12.88	110.84 ± 5.16
138.17 ± 29.41	
181.80 ± 6.80	
115.42 ± 7.70	
168.33 ± 30.21	
118.37 ± 9.60	
137.26 ± 5.00	
Mean: 145.04 ± 24.50	Mean: 130.08 ± 16.84
p = 0.13	

AT: atrial tachycardia.
